# Typical Gaseous Semi-Volatile Metals Adsorption by Meta-Kaolinite: A DFT Study

**DOI:** 10.3390/ijerph15102154

**Published:** 2018-09-30

**Authors:** Xinye Wang, Min Chen, Changqi Liu, Changsheng Bu, Jubing Zhang, Chuanwen Zhao, Yaji Huang

**Affiliations:** 1 Jiangsu Provincial Key Laboratory of Materials Cycling and Pollution Control, School of Energy and Mechanical Engineering, Nanjing Normal University, Nanjing 210042, China; chenminszx@163.com (M.C.); csbu@njnu.edu.cn (C.B.); jubingzhang@njnu.edu.cn (J.Z.); 2 Key Laboratory of Energy Thermal Conversion and Control of Ministry of Education, School of Energy and Environment, Southeast University, Nanjing 210096, China; changqiliu@foxmail.com (C.L.); heyyj@seu.edu.cn (Y.H.)

**Keywords:** lead, cadmium, sodium, potassium, kaolinite, adsorption

## Abstract

Kaolinite can be used as in-furnace adsorbent to capture gaseous semi-volatile metals during combustion, incineration, or gasification processes for the purposes of toxic metals emission control, ash deposition/slagging/corrosion inhibition, ultrafine particulate matter emission control, and so on. In this work, the adsorptions of typical heavy metals (Pb and Cd) and typical alkali metals (Na and K) by meta-kaolinite were investigated by the DFT calculation. The adsorption energies followed the sequence of NaOH-Si surface > KOH-Si surface > PbO-Al surface ≈ CdO-Al surface ≈ NaOH-Al surface > KOH-Al surface > NaCl-Al surface ≈ Na-Si surface > Na-Al surface > KCl-Al surface > Pb-Al surface > PbCl_2_-Al surface > CdCl_2_-Al surface ≈ K-Si surface ≈ PbCl-Al surface > K-Al surface > CdCl-Al surface > NaCl-Si surface > KCl-Si surface > Cd-Al surface. Si surface was found available to the adsorptions of Na, K, and their compounds, although it was invalid to the adsorptions of Pb, Cd, and their compounds. The interactions between adsorbates and surfaces were revealed. Furthermore, the discussion of combining with the experimental data was applied to the subject validity of calculation results and the effect of chlorine on adsorption and the effect of reducing atmosphere on adsorption.

## 1. Introduction

As a nonmetallic mineral, kaolinite is widely distributed in the world with large proven reserves. Its global production was 37 million tons in 2017 [1]. The primary uses of kaolinite are paper coating and filling, ceramics, paint, refractories, etc. due to its whiteness, brightness, glossiness, abrasiveness, viscosity, and fine particle size [2]. Besides the traditional uses, kaolinite can be used as in-furnace adsorbent to capture gaseous semi-volatile metals during combustion, incineration, or gasification processes for several purposes [3,4,5].

The first purpose is to control submicron heavy metals emission during coal, municipal solid waste, or sewage sludge combustion. Semi-volatile metals such as lead (Pb) and Cadmium (Cd) evaporate partially in furnace, and then condense in flue to be submicron aerosols (PM_1_) which are difficult to remove efficiently via an electrostatic precipitator and bag filter [6,7,8,9]. Furthermore, activated carbon cannot remove these fine particles effectively either, although it is effective to remove elemental mercury which remains gaseous at low temperature [10]. The addition of supermicron kaolinite powder into the furnace can induce semi-volatile metals migration from submicron aerosols to supermicron particles [5,11,12,13]. Yao et al. added 5% of kaolinite in dried sludge, resulting in capture efficiencies of 50% and 40% for the control of submicron Pb and submicron Cd emissions, respectively, during the drop tube furnace combustion [11].

The second purpose is to lessen ash deposition, slagging and high-temperature corrosion during high potassium (K) biomass and high sodium (Na) coal combustion or some co-combustion situations. These alkali metals volatilize in furnace, then react with ash particles to form low melting point phases rendering the particle surfaces sticky, or form sulfate condensing on heating surfaces directly [3,14]. A similar situation occurs in bed agglomeration, causing the defluidization of fluidized beds [15]. Aho added kaolinite in a 20 kW bubbling bed reactor with the fuel of biomass, and no coating of bed particles occurred at dosage >0.3 times the mass flow of ash [16].

The third purpose is to reduce particulate matter emissions during coal combustion, which contain high levels of alkali metals. Na and K have been proven to contribute greatly to ultrafine particulate matters formation by vaporization and condensation during the combustion of high Na coal and biomass, respectively [17,18]. Sun et al. added 3% raw kaolinite and modified kaolinite into pulverized coal and reduced by about 16% and 40% PM_0.2_ respectively during combustion in drop tube furnace [18]. The fourth purpose is to mitigate the blade corrosion in gas turbine of integrated gasification combined cycle (IGCC) and pressurized fluidized-bed combustors (PFBC) in combined-cycle power generation systems [19,20].

The effect of kaolinite on metal capture has been investigated extensively, however, few studies focused on the capture mechanisms which has not been understood well yet [5,13]. Wendt et al. developed the aerosol size fractionation method (ASFM) to investigate the effect and mechanisms of Pb/Cd/Na capture by kaolinite powder with in-furnace injection process as industrial application background [21]. They described the kinetics of single metal capture and dual metals capture [12,22]. Shadman et al. developed thermogravimetric on-line detect method to investigate the effect and mechanisms of Pb/Cd/Na capture by kaolinite flake with high temperature fixed bed process as an industrial application background [23,24,25]. The resultant data included reaction product, saturated adsorption amount, and morphology changes [23,24,25,26].

Most previous research attempted to understand the capture mechanisms by experiments and obtained the results at macroscopic level. Some important phenomena and effects were found and interpreted [12]. However, the investigation of the capture mechanisms of heavy metals and alkali metals by kaolinite at the atomic level was just started. Since the capture occurs above 800 °C usually, it was difficult to conduct the atomic level observation of capture processes. The theoretical calculation based on density functional theory (DFT) was considered as an appropriate method to investigate these processes at an atomic level at the present stage. In the previous research, we calculated the adsorption of Pb and Cd on kaolinite surfaces, and revealed some information on adsorption sites, the adsorption energies and so on [27,28]. Recently, the calculation of Na adsorption was reported as well [29]. Many experimental phenomena were interpreted by the results from calculation, for example, the difference between Pb capture and Cd capture [27].

In this work, further investigation on the capture mechanisms was carried out. The calculation objects contained not only heavy metals (Pb and Cd) but also alkali metals (Na and K). Meta-kaolinite was considered as the adsorbent for the dehydroxylation of kaolinite caused by the flash calcination in the furnace [30]. This extensive comparison of different adsorption cases would give more interpretation and prediction to the results from experiment and application. Besides, a deeper understanding of adsorption mechanisms would help to figure out how to improve the capture ability of kaolinite.

## 2. Modeling and Computational Details

### 2.1. Adsorbates

**Figure 1:**
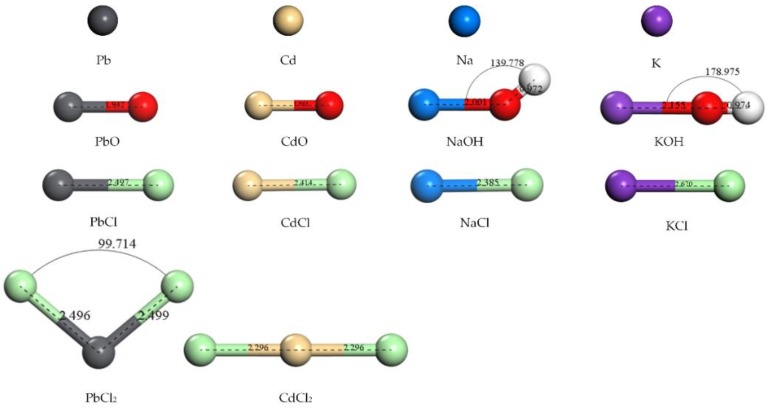
Geometric structures of adsorbates. The units of the bond length and bond angles are Å and °, respectively.

The species of heavy metals and alkali metals in furnace were difficult to detect by experiments, instead, they were predicted by thermodynamic equilibrium calculation based on Gibbs free energy minimization. It was found that the major species of Pb were monoxide, dichloride, monochloride, and single atoms in a waste incinerator while the major species of Na and K were considered as monochloride and hydroxide in the furnace [31,32]. These species were also involved in the combustion process and gasification process of coal or biomass [33,34,35]. In this work, the species of adsorbates involved in the calculation contained the single atoms of Pb, Cd, Na, and K; the oxides of PbO and CdO; the hydroxides of NaOH and KOH; the monochlorides of PbCl, CdCl, NaCl, and KCl; and the dichlorides of PbCl_2_ and CdCl_2_. The original structures of these adsorbates were downloaded from NIST Chemistry WebBook [36]. Then, these original structures were optimized in the lattice by DFT calculation. The computational details are described in Section 2.3. The optimized structures of adsorbates are shown in Figure 1.

The structures of the diatomic molecules in Figure 1 are similar to each other, for example, the bond lengths of PbO and CdO are around 1.9 Å; and the bond lengths of PbCl, CdCl, NaCl, and KCl are around 2.4 Å. However, the structures of the triatomic molecules in Figure 1 are different from each other, for example, NaOH and PbCl_2_ are broken line molecules while KOH and CdCl_2_ are linear molecules. The difference in structures should cause differences in adsorption characteristics.

### 2.2. Adsorption Surfaces

**Figure 2:**
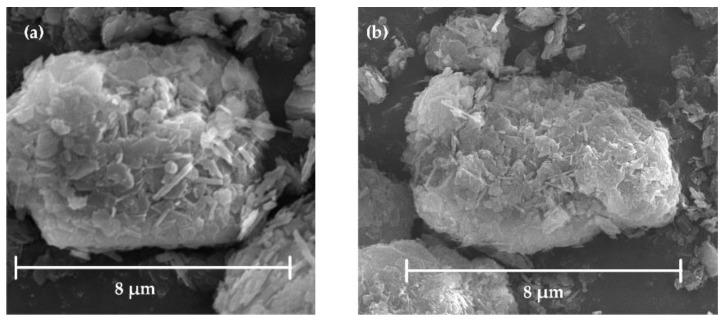
SEM micrographs of (**a**) raw kaolinite and (**b**) calcined kaolinite at 1000 °C for 30 min.

The raw kaolinite and calcined kaolinite were detected by scanning electron microscope (SEM) and the results are shown in Figure 2a,b. Both the raw kaolinite particle and calcined kaolinite were composed of the stacked flakes. The surface morphology of these flakes suggested that the layer structure remained well after the dehydroxylation induced by calcination. Therefore, the upper and lower surfaces of layers should dominate the total exposed surfaces, and the side surfaces were minority and negligible. We have investigated the adsorption ability of upper and lower surfaces which were described as aluminum (Al) surface (0 0 1) and silicon (Si) surface (0 0 $\bar{1}$) [27,28]. Al surface has the abilities of Pb and Cd adsorption while an Si surface does not during the research on Pb and Cd adsorption [27,28]. However, it was unknown if Si surface was invalid in Na and K adsorption either. Consequently, both Al and Si surfaces were investigated as adsorption surfaces in this work.

**Figure 3:**
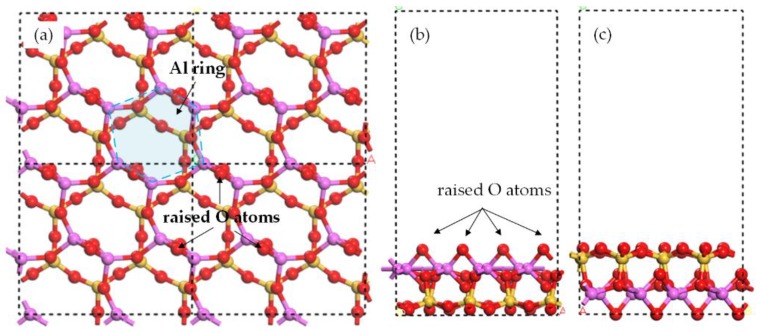
Optimized structure of 2 × 2 Al surface. (**a**) Top view of Al surface, (**b**) side view of Al surface, and (**c**) side view of Si surface. Yellow ball = Si atom, purple ball = Al atom, red ball = O atom, and white ball = H atom.

The Al/Si surface of meta-kaolinite was derived from the Al/Si surface of kaolinite by the method of gradual dehydroxylation described before in [28]. Considering the weak interaction between layers of meta-kaolinite, only one layer was used as sorbent. The test of the layer number supported this decision that the difference between the adsorption energies of one-layer and two-layer structures was less than 2%. The supercell size was 2 × 1 × 1 and the vacuum thickness was 15 Å. The structures of meta-kaolinite surface are shown in Figure 3. No atoms were constrained during geometry optimization.

### 2.3. Computational Methods

The DFT calculations were on the basis of the plane-wave pseudopotential approach (CASTEP program) with the Vanderbilt ultra-soft pseudopotential, the generalized gradient approximation and the Perdew, Burke, and Ernzerhof functional (GGA-PBE) [37,38]. The geometry optimization algorithm was Broyden-Fletcher-Goldfarb-Shanno (BFGS) [39]. The plane-wave basis set energy cutoff was set at 380 eV. The convergence parameters were 5 × 10^−7^ eV/atom for self-consistent field (SCF) tolerance, 5 × 10^−6^ eV/atom for energy, 0.01 eV/Å for maximum force, 0.02 GPa for maximum stress, and 5 × 10^−4^ Å for maximum displacement. The adsorption energy (Δ*E*_adsorption_) was defined as Equation (1).

Δ*E*_adsorption_ = *E*_sorbate+sorbent_ − (*E*_sorbate_ + *E*_sorbent_) (1)

where *E*_sorbate+sorbent_, *E*_sorbate_, and *E*_sorbent_ are the energies of the sorbent with the adsorbed molecule, the sorbate, and bare sorbent, respectively. The charge density difference (Δ*ρ*) was defined as Equation (2).

Δ*ρ* = *ρ*_sorbate+sorbent_ − (*ρ*_sorbate_ + *ρ*_sorbent_) (2)

where *ρ*_sorbate+sorbent_, *ρ*_sorbate_, and *ρ*_sorbent_ are the electron densities of the sorbent with the adsorbed molecule, the sorbate, and bare sorbent, respectively. Mulliken population was calculated for the analysis of charge transfer behavior [40].

## 3. Results

### 3.1. Single Atom Adsorption on Al Surface

**Figure 4:**
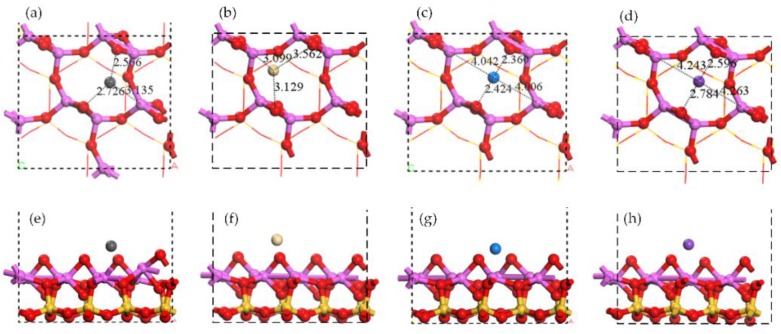
Optimized structures of single atom adsorptions on Al surface. Top view of (**a**) Pb adsorption, (**b**) Cd adsorption, (**c**) Na adsorption, and (**d**) K adsorption. Side view of (**e**) Pb adsorption, (**f**) Cd adsorption, (**g**) Na adsorption, and (**h**) K adsorption.

The structures of single atom adsorption on Al surface are shown in Figure 4. The stable adsorption site with the highest adsorption energy was close to the center of Al ring. The Pb atom was close to two raised oxygen (O) atoms and one Al atom in Al surface. The position of Cd atom was similar to that of Pb atom, however, the Cd atom was closer to the Al atom and farther away from two raised O atoms. The Na atom and the K atom were almost on the center of Al ring. Compared with the K atom, the Na atom seemed to have stronger attraction to the two raised O atoms, because the distances of Na-O (around 2.4 Å) were shorter than that of K-O (around 2.6 Å).

**Figure 5:**
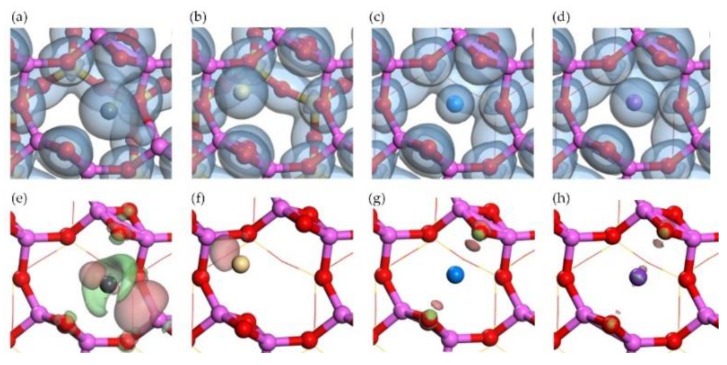
Electron density of single atom adsorptions on Al surface: top view of (**a**) Pb adsorption, (**b**) Cd adsorption, (**c**) Na adsorption, and (**d**) K adsorption. Electron density difference of single atom adsorptions on Al surface: top view of (**e**) Pb adsorption, (**f**) Cd adsorption, (**g**) Na adsorption, and (**h**) K adsorption. Pink means charge accumulation while green means charge depletion.

The distributions of electron density and electron density difference are shown in Figure 5. The interaction between the Pb atom and the Al atom was obvious, while the interaction between the Cd atom and Al atom existed as well but was weaker. No similar interaction was found in Na adsorption or K adsorption. The charge accumulation between a single atom and two raised O atoms was close to the Pb atom but was away from the Na atom and the K atom. It indicated that Na-O has a stronger ionic bond characteristic than Pb-O which exhibited a covalent bond characteristic [41]. The interaction between the K atom and two raised O atoms was weak and no interaction was found between the Cd atom and two raised O atoms.

The adsorption energies and the Mulliken charge change were listed in Table 1. The adsorption energies followed the sequence of Na > Pb > K > Cd. The first three adsorptions (Na, Pb, and K) were chemical while the last (Cd) was physical judging from the adsorption energies. The Mulliken charge changes show the similar sequence but did not coincide completely. It is noteworthy that the interaction between single atom and two raised O atoms in Al surface followed the same sequence to adsorption energies. It presented in the distance and charge accumulation described above. For example, *d*_Na__-O_ = 2.39 Å < *d*_Pb-O_ = 2.65 ≈ *d*_K-O_ = 2.69 Å < *d*_Cd-O_ = 3.35 Å, where *d* indicates the average distance between the metal atom and two raised O atoms. Therefore, the attraction from the raised O atoms was considered to play the dominant role in single atom adsorption on Al surface while the attraction from Al atoms was less important.

### 3.2. Oxide and Hydroxide Adsorption on Al Surface

**Figure 6:**
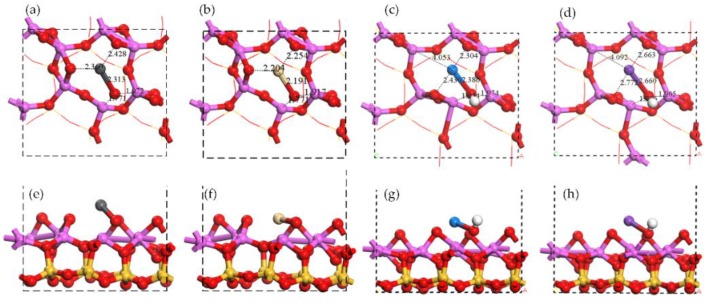
Optimized structures of oxide molecule adsorptions and hydroxide molecule adsorptions on Al surface. Top view of (**a**) PbO adsorption, (**b**) CdO adsorption, (**c**) NaOH adsorption, and (**d**) KOH adsorption. Side view of (**e**) PbO adsorption, (**f**) CdO adsorption, (**g**) NaOH adsorption, and (**h**) KOH adsorption.

On the Al surface, the Al ring consists of six sides of Al-O-Al. Two raised O atoms are on the opposite sides as shown in Figure 3. The adsorption of PbO molecule caused the move of one raised O atom to the side next to its original site and far away from the O atom in PbO, as shown in Figure 6a. In the adsorption structure, the Pb atom was in the center of Al ring and the O atom in PbO seemed to be the third raised O atom in Al ring, forming a new symmetrical structure. The lengths of two newly formed Al-O bonds (the O atom was from PbO) were 1.77 Å and 1.97 Å, close to the original bond length of 1.76 Å in Al surface. The PbO molecule was elongated so that the distances between the Pb atom and three raised O atoms were all around 2.3 Å, resulting in the center site of Pb atom.

The adsorption structure of CdO molecule was pretty similar to that of PbO molecule, as shown in Figure 6b. However, the structures of NaOH adsorption and KOH adsorption were different. Although the position of the Na atom was also in the center of Al ring, and the O atom in NaOH seemed to bond with the Al atoms, the raised O atom originally in Al surface did not move to other sites, as shown in Figure 6c. Therefore, the adsorption structure of the NaOH molecule was not as symmetrical as that of PbO molecule. Because of the attractions from the surface to the O atom from NaOH, the bond length of Na-O in NaOH was elongated as well. The structure of the KOH molecule is linear before adsorption; however, it turned into a broken line type like the NaOH molecule after adsorption.

**Figure 7:**
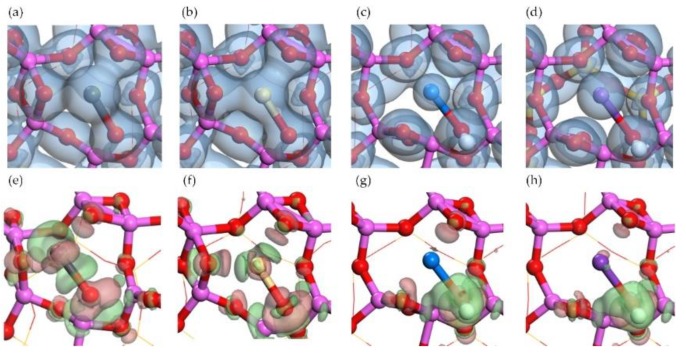
Electron density of oxide molecule adsorptions and hydroxide molecule adsorptions on Al surface: top view of (**a**) PbO adsorption, (**b**) CdO adsorption, (**c**) NaOH adsorption, and (**d**) KOH adsorption. Electron density difference of oxide molecule adsorptions and hydroxide molecule adsorptions on Al surface: top view of (**e**) PbO adsorption, (**f**) CdO adsorption, (**g**) NaOH adsorption, and (**h**) KOH adsorption.

The electron density distribution shown in Figure 7a described the overlap of electron density between the Pb atom and three raised O atoms, one of which was derived from PbO. The symmetric shape of isosurface suggested that the O atom derived from PbO was integrated into Al surface so well that it exhibited few differences from two original raised O atoms. Moreover, this overlap indicated the covalent characteristic of three Pb-O bonds, which were supported by the charge accumulation between the Pb atom and two raised O atoms in Figure 7e [41]. No overlap of electron density between the O atom derived from PbO and Al atoms and no charge accumulation between them, but the charge accumulation around the O atom derived from PbO. It indicated the ionic characteristic of an O-Al bond, same as the Al_2_O_3_ crystal. The Mulliken charge changes in Table 1 indicated the charge acceptance of the O atom derived from PbO as well. The same characteristics were found in CdO adsorption structure by the analysis of Figure 7b,f. However, in NaOH adsorption and KOH adsorption, no overlap of electron density between Na-O or K-O were found and the charge accumulated around raised O atoms as shown in Figure 7g,h, indicating the ionic characteristics of Na-O bond and K-O bond. Besides that, the O-H derived from NaOH or KOH was considered as the charge donator to Al surface according to the analysis of electron density difference and the Mulliken charge change. Overall, the interactions between the O atoms derived from sorbates and the Al atoms were stronger than the interactions between the metal atoms derived from sorbates and the raised O atoms.

The adsorption energies of PbO, CdO, NaOH, and KOH shown in Table 1 were much higher than that of single atoms and followed the sequence of PbO ≈ CdO ≈ NaOH > KOH. These adsorptions were all chemical for the adsorption energies higher than −350 kJ/mol. Although the cations of four compounds were different, their adsorption energies were very close. It should be due to the strong attraction between the O atom and the Al atoms in the adsorption structure. The interaction between the metal atom and the raised O atoms did not dominate the adsorption.

### 3.3. Chloride Adsorption on Al Surface

**Figure 8:**
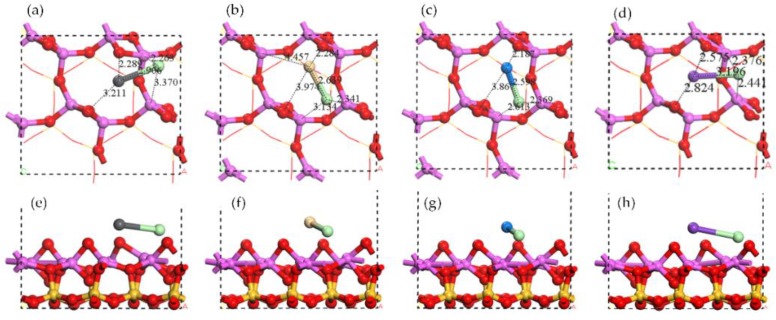
Optimized structures of monochloride molecule adsorptions on Al surface. Top view of (**a**) PbCl adsorption, (**b**) CdCl adsorption, (**c**) NaCl adsorption, and (**d**) KCl adsorption. Side view of (**e**) PbCl adsorption, (**f**) CdCl adsorption, (**g**) NaCl adsorption, and (**h**) KCl adsorption.

The adsorption structures of monochlorides are shown in Figure 8. In PbCl adsorption structure the Pb atom was on the center of Al ring while the Cl atom was close to two Al atoms. The similar structure was shown in the adsorption of CdCl, NaCl, and KCl. The difference was in the average distance of Cl-Al that *d*_K__-Cl_ = 2.41 ≈ *d*_Na__-Cl_ = 2.49 Å < *d*_Cd__-Cl_ = 2.74 Å ≈ *d*_Pb__-Cl_ = 2.82 Å. All four monochloride molecules were elongated for adsorption.

**Figure 9:**
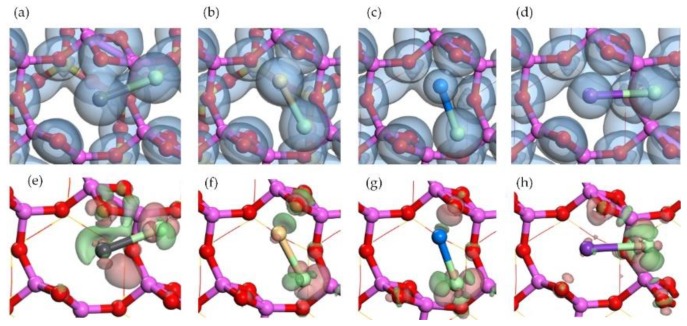
Electron density of monochloride molecule adsorptions on Al surface: top view of (**a**) PbCl adsorption, (**b**) CdCl adsorption, (**c**) NaCl adsorption, and (**d**) KCl adsorption. Electron density difference of monochloride molecule adsorptions on Al surface: top view of (**e**) PbCl adsorption, (**f**) CdCl adsorption, (**g**) NaCl adsorption, and (**h**) KCl adsorption.

The distribution of electron density and electron density difference of monochloride adsorptions are shown in Figure 9. The charge accumulation was found between the Cl atom and the Al atoms, indicating the covalent characteristic of Cl-Al in all four adsorption structures. There was obvious charge accumulation between the Pb atom and the Al atom in PbCl adsorption, the same as that in Pb atom adsorption. Moreover, no such interaction was found in the adsorption of other monochlorides, the same as the in single adsorption as well. The Mulliken charge changes of the Cl atom in PbCl adsorption and CdCl adsorption were much lower than that in NaCl adsorption and KCl adsorption. Compared with the interaction between the Cl atom and the Al atoms, the interaction between metal atom and the raised O atom was insignificant. The adsorption energies sequence of NaCl > KCl > PbCl > CdCl shown in Table 1 indicated that the Cl atom bonding with Na and K was more active than that bonding with Pb and Cd.

**Figure 10:**
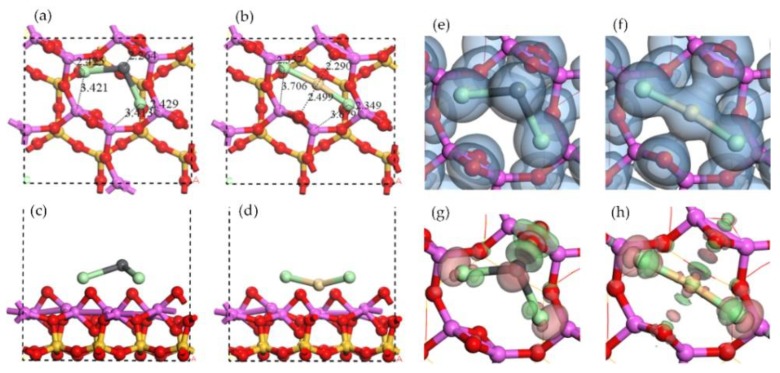
Optimized structures of dichloride molecule adsorptions on Al surface. Top view of the structures of (**a**) PbCl_2_ adsorption and (**b**) CdCl_2_ adsorption. Side view of the structures of (**c**) PbCl_2_ adsorption and (**d**) CdCl_2_ adsorption. Electron density difference of dichloride adsorption structures on Al surface: top view of (**e**) PbCl_2_ adsorption, (**f**) CdCl_2_ adsorption. Side view of (**g**) PbCl_2_ adsorption, and (**h**) CdCl_2_ adsorption.

The dichloride adsorption structures are shown in Figure 10a–d. The PbCl_2_ molecule has the shape of a broken line. In Figure 10a,c, the Cl atom was above Al-O-Al and was close to one of Al atom. The Pb atom was close to one raised O atom. The CdCl_2_ molecule has the shape of straight line. In Figure 10b,d, the positions of Cl atom were similar to that of PbCl_2_ adsorption, but was closer one of Al atom in Al-O-Al. The Cd atom was almost on the center of the Al ring. The distribution of electron density and electron density difference of dichloride adsorption structures are shown in Figure 10e–h. The overlap of electron density between the Pb atom and one raised O atom and the charge accumulation between the Cl atoms and the Al atoms indicated the covalent characteristic of Pb-O bond and Cl-Al bond. The similar bond characteristics were shown in CdCl_2_ adsorption structure. According to Table 1, the adsorption energies of PbCl_2_ (−209 kJ/mol) and CdCl_2_ (−199 kJ/mol) increased with the additional Cl atoms compared with those of PbCl (−196 kJ/mol) and CdCl (−149 kJ/mol), however, these increases were limited.

### 3.4. Alkali Metal Adsorption on Si Surface

**Figure 11:**
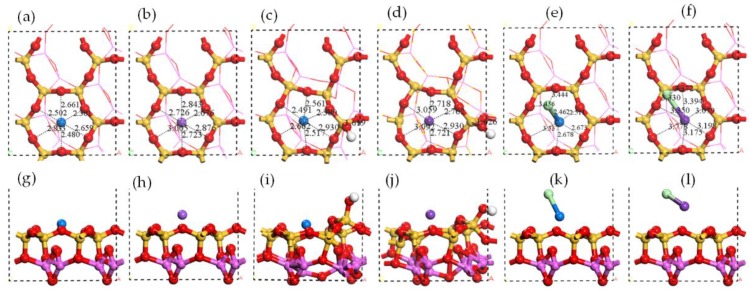
Optimized structures of alkali metal adsorptions on Si surface. Top view of the structures of (**a**) Na atom adsorption, (**b**) K atom adsorption, (**c**) NaOH adsorption, (**d**) KOH adsorption, (**e**) NaCl adsorption, and (**f**) KCl adsorption. Side view of the structures of (**g**) Na atom adsorption, (**h**) K atom adsorption, (**i**) NaOH adsorption, (**j**) KOH adsorption, (**k**) NaCl adsorption, and (**l**) KCl adsorption.

**Figure 12:**
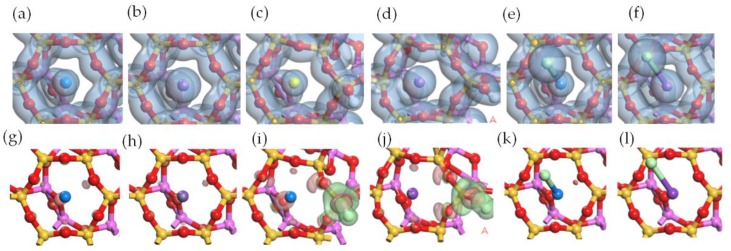
Electron density of single atom adsorptions on Si surface: top view of (**a**) Na atom adsorption, (**b**) K atom adsorption, (**c**) NaOH adsorption, (**d**) KOH adsorption, (**e**) NaCl adsorption, and (**f**) KCl adsorption. Electron density difference of single atom adsorptions on Al surface: top view of (**g**) Na atom adsorption, (**h**) K atom adsorption, (**i**) NaOH adsorption, (**j**) KOH adsorption, (**k**) NaCl adsorption, and (**l**) KCl adsorption.

Si surface was considered invalid to the adsorption of Pb, Cd, and their compounds [27,28]. However, it showed ability to adsorb Na, K, and their compounds. The adsorption structures of single atoms, hydroxide, and monochloride of Na and K on Si surface are shown in Figure 11. The Na atom and K atom were considered to be adsorbed on the center of Si ring other than on the top of O atom according to the comparison of adsorption energies. The charge transfers after the adsorption of the Na atom and the K atom on Si surface were higher than that on Al surface, as shown in Table 1 and Table 2. So were the adsorption energies, which indicated that the adsorptions of Na atom and K atom were chemical. The charge accumulation was found not only between the adsorbed atom and the O atoms from Si ring but also between the adsorbed atom and one O atom from Al ring, as shown in Figure 12a,b,g,h. The adsorptions of NaOH and KOH caused the decomposition of metal atom and OH. The adsorption structures of NaOH and KOH in Figure 12c,d indicated that the alkali atom remained on the center of Si ring while OH combined with the Si atom causing the broken of Si-O-Al. The adsorption energies of NaOH and KOH on Si surface were higher than −600 kJ/mol, the highest in all cases. The distribution of electron density difference in Figure 12i,j revealed the strong interaction between OH and the Si atom, which was considered as the reason for the high adsorption energies of NaOH and KOH. According to the adsorption energy in Table 2, NaCl was adsorbed chemically while KCl was physically. The distribution of electron density difference in Figure 12k,l indicated that the Na atom had interaction with the O atoms from Si ring and Al ring, the K atom had no obvious interaction with surface and the Cl atom had no interaction with surface. Therefore, the attraction between NaCl/KCl and Si surface was due to the interaction between the Na/K atom and surface. The Cl atom in NaCl and KCl inhibited the adsorption on Si surface, however, it was in favor of the adsorption on Al surface. The adsorption energies in Table 2 were higher than those obtained by Zhang et al. [29].

## 4. Discussion

### 4.1. Comparison with Experimental Data

The adsorption of heavy metals and alkali metals at high temperature consists of not only the surface adsorption but also the inward diffusion [27,29]. No experimental data could be used to compare with calculation data directly. Therefore, the comparison of the experimental data and the calculation data were discussed conservatively. The experimental studies on the metal oxide and hydroxide adsorption by kaolinite were carried out by Wendt et al. who developed the aerosol size fractionation method (ASFM) to detect the reactions between PbO/CdO/NaOH and kaolinite powder in an 18 kW vertical combustor [12,21,22,42]. The capture efficiency of PbO vapor was close to that of NaOH vapor and higher than that of CdO vapor at 1160 °C; however, all these efficiencies turned to close to each other at 1280 °C [12,22,42]. Wendt et al. attributed the difference between PbO/NaOH adsorption and CdO adsorption at lower temperatures to the products melting which helped the diffusion of products to inside of kaolinite and the exposure of unreacted kaolinite to outside [12,42]. The products of PbO adsorption and NaOH adsorption melted at 1160 °C but the products of CdO adsorption did not melt until the temperature was up to 1280 °C [12,42]. According to the adsorption energies in this work, there was no obvious difference in the adsorption of PbO, CdO, and NaOH. Therefore, the adsorptions of PbO, CdO, and NaOH should have less difference in chemical process but more in physical process. Yao et al. compared the capture of PbCl_2_ and CdCl_2_ by kaolinite during sewage sludge combustion [43]. At 800–950 °C, the capture efficiencies of PbCl_2_ was higher than that of CdCl_2_, agreeing with the higher adsorption energy of PbCl_2_ than that of CdCl_2_ in this work. In this view, it is possible to use adsorption energy to evaluate the chemical adsorption process of heavy metals and alkali metals vapor by kaolinite.

It is a pity that no available data about the adsorption of K and its compounds were found so that the discussion combining with experimental data and calculation data was absent here. In this work, the adsorption energies of K and its compounds were always lower than that of Na and its compounds. Therefore, it is inferred that the adsorption efficiencies of K and its compound would be weaker than that of Na and its compounds as well.

### 4.2. Effect of Chlorine Metal Capture

The thermodynamic equilibrium studies indicated that chloride was the main species of Pb, Cd, Na, and K in furnace at around 1000 °C when the solid fuel contained sufficient Cl [31]. Wendt et al. found that the addition of Cl depressed the metal capture by kaolinite significantly. Therefore, chloride was more difficult to absorb than oxide or hydroxide by kaolinite. The much lower adsorption energies of chlorides than those of oxides and hydroxide in this work supported this experimental conclusion. Moreover, there was no Cl in the products of metal chloride adsorption, so the process of Cl release should follow the chloride adsorption [3,23,29]. The lower adsorption energy and the Cl release process would be the main reason for low capture efficiency of chlorides. According to the thermodynamic equilibrium calculation, higher temperature causes less metal chlorides and more metal oxides during combustion [31]. Therefore, the high temperature can enhance the metal capture by kaolinite through not only the increase of reaction rate and the promotion of products melting but also the transformation of chlorides to oxides.

### 4.3. Effect of Reducing Atmosphere on Metal Capture

The atmosphere mentioned here indicates the oxidizing atmosphere and the reducing atmosphere. In the reducing atmosphere, some heavy metals such as Pb and Cd were supposed to be the elementary substance vapor according to the experimental research and the thermodynamic equilibrium calculation [4,31]. The metal adsorption in reducing atmosphere and the elementary metal vapor adsorption by kaolinite has not been reported yet. The calculation results in this work indicated that Pb vapor adsorption was possible because the Pb atom adsorption energy was as high as −224 kJ/mol, however, Cd vapor adsorption was impossible because the adsorption energy was physical. During the combustion, some local areas in furnace are at reducing atmosphere, moreover, the solid fuel inside or surrounding are at reducing atmosphere. Thus, the presence of elementary Pb vapor was possible and a new adsorption path was proposed. During combustion, kaolinite adsorbed elementary Pb vapor first at reducing atmosphere, and then the adsorption was oxidized in an oxidizing atmosphere, turning to PbO adsorption which was much more stable than elementary Pb adsorption.

### 4.4. Competitive Adsorption of Multicomponents

Gale et al. found that PbO and CdO could improve each other’s capture efficiency by enhancing the eutectic-melt during CdO adsorption by kaolinite and avoiding the excessive-melt during PbO adsorption by kaolinite [44]. According to similar adsorption energy of PbO and CdO, the competition between their adsorption would be equal in chemistry. Gale et al. found that the presence of NaOH inhibited PbO capture and the presence of PbO had no effect on NaOH adsorption [44]. They considered NaOH to have a slightly higher reaction rate than PbO, thus achieving capture before the most significant sorbent deactivation occurs [44]. The calculation results supported this opinion that NaOH could adsorb on both Al surface and Si surface while PbO could only adsorb on Al surface. The excessive melt should be induced by NaOH mainly. Further investigation should be performed to reveal mechanisms of the competitive adsorption.

## 5. Conclusions

The adsorptions of typical heavy metals (Pb and Cd) and typical alkali metals (Na and K) by meta-kaolinite were investigated by the DFT calculation. The adsorption energies were obtained and followed the sequence of NaOH-Si surface > KOH-Si surface > PbO-Al surface ≈ CdO-Al surface ≈ NaOH-Al surface > KOH-Al surface > NaCl-Al surface ≈ Na-Si surface > Na-Al surface > KCl-Al surface > Pb-Al surface > PbCl_2_-Al surface > CdCl_2_-Al surface ≈ K-Si surface ≈ PbCl-Al surface > K-Al surface > CdCl-Al surface > NaCl-Si surface > KCl-Si surface > Cd-Al surface. All the adsorptions on Al surface were chemical except for the Cd atom adsorption. The adsorptions of metal atoms were due to the attraction from the raised O atoms in Al surface. The adsorptions of metal oxide and hydroxide were due to the attraction from the Al atoms to the O atom. The adsorptions of metal chloride were due to the attraction from the Al atoms to the Cl atoms. Although Pb, Cd, and their compounds did not adsorb on Si surface, Na, K, and their compounds did. The Cl atom in NaCl and KCl inhibited adsorption on Si surface; however, it was in favor of adsorption on Al surface. The strong adsorptions of NaOH and KOH were due to their decomposition and the products of OH.

## Figures and Tables

**Table 1:** Mulliken charge changes and adsorption energies of 14 species adsorptions on Al surface.

Sorbate	Mulliken Charge Change ^1^ (e)				Adsorption Energy (kJ/mol)
Pb	Pb: +0.49			Total: +0.49	−224
Cd	Cd: +0.22			Total: +0.22	−36
Na	Na: +0.70			Total: +0.70	−243
K	K: +0.79			Total: +0.79	−168
PbO	Pb: +0.42	O: −0.40		Total: +0.02	−476
CdO	Cd: +0.43	O: −0.35		Total: +0.08	−471
NaOH	Na: +0.14	O: +0.18	H: +0.02	Total: +0.34	−474
KOH	K: +0.14	O: +0.30	H: +0.01	Total: +0.45	−368
PbCl	Pb: +0.18	Cl: −0.02		Total: +0.16	−196
CdCl	Cd: +0.10	Cl: +0.02		Total: +0.12	−149
NaCl	Na: +0.13	Cl: +0.25		Total: +0.38	−300
KCl	K: +0.15	Cl: +0.39		Total: +0.54	−239
PbCl_2_	Pb: +0.1	Cl1: 0	Cl2: 0	Total: +0.10	−209
CdCl_2_	Cd: +0.05	Cl1: +0.06	Cl2: +0.06	Total: +0.16	−199

^1^ Mulliken charge change is defined as the charge difference after and before adsorption. + indicates electron donation while − indicates electron acceptance.

**Table 2:** Mulliken charge changes and adsorption energies of six species adsorption on Si surface.

Sorbate	Mulliken Charge Change (e)				Adsorption Energy (kJ/mol)
Na	Na: +1.09			Total: +1.09	−295
K	K: +1.17			Total: +1.17	−197
NaOH	Na: +0.42	O: +0.15	H: +0.11	Total: +0.68	−742
KOH	K: +0.35	O: +0.31	H: +0.05	Total: +0.71	−655
NaCl	Na: +0.13	Cl: +0.05		Total: +0.18	−142
KCl	K: +0.17	Cl: +0.09		Total: +0.21	−52
